# Social Support and Postpartum Depressive Symptoms in Portuguese Women: The Mediating Role of Emotion Regulation Difficulties

**DOI:** 10.3390/jcm13237150

**Published:** 2024-11-26

**Authors:** Tânia Brandão, Ana Catarina Ribeiro, Maria Inês Griff, Alessandra Babore, Eva Diniz

**Affiliations:** 1 William James Center for Research, Ispa—Instituto Universitário, Rua Jardim do Tabaco, 44, 1149-041 Lisboa, Portugal; ediniz@ispa.pt; 2 School of Psychology, Ispa—Instituto Universitário, Rua Jardim do Tabaco, 44, 1149-041 Lisboa, Portugal; anacatarinagr2000@gmail.com (A.C.R.); inesgriff@gmail.com (M.I.G.); 3 Department of Psychology, University “G. d’Annunzio” via dei Vestini, 31, 66100 Chieti, Italy; a.babore@unich.it

**Keywords:** social support, emotion regulation difficulties, postpartum depressive symptoms

## Abstract

**Background/Objectives:** Postpartum depression (PPD) is a prevalent mental health issue affecting 14% of mothers worldwide, with long-term implications for both maternal and child well-being. Understanding the factors contributing to PPD is essential for developing effective interventions. This study aimed to investigate the relationship between social support and postpartum depression symptoms, with a focus on the mediating role of emotion regulation difficulties. **Methods:** A sample of 160 postpartum women (M age = 33.57, SD = 4.94) participated in the study. Participants were assessed on their levels of perceived social support, difficulties in emotion regulation, and symptoms of PPD. **Results:** The results indicated that lower levels of social support were significantly associated with greater difficulties in emotion regulation (effects ranging from −0.10 to 0.07). These difficulties in turn were linked to higher levels of postpartum depression symptoms (effects ranging from −0.29 to 0.78), suggesting a partial mediation effect from emotional awareness (95% CI −0.05, −0.00), non-acceptance of emotions (95% CI −0.04, −0.00), difficulty in goal-directed behavior (95% CI −0.04, −0.00), and limited access to strategies (95% CI −0.12, −0.04). **Conclusions:** These findings underscore the critical role of both social support and emotion regulation in the development of PPD symptoms. Enhancing emotion regulation skills, particularly for women with limited social support, could be a key target for interventions aimed at reducing the risk and severity of PPD.

## 1. Introduction

Postpartum depression (PPD) is characterized by a range of symptoms, including persistent sadness, loss of interest in activities, feelings of hopelessness, and difficulty bonding with the newborn [1,2]. Unlike the transient “*baby blues*”, which usually subside within two weeks after childbirth, PPD is a more severe and prolonged condition, lasting up to one year postpartum and affecting approximately 14% of mothers worldwide [2,3,4]. Given its prevalence and the potential long-term consequences for the child (e.g., impaired language and personal–social development) [5,6], the mother (e.g., increased risk of suicide) [7,8], and even the father (e.g., heightened parental stress) [7], understanding the factors that contribute to the onset and persistence of PPD has become a critical public health priority.

The etiology of PPD is multifactorial, involving complex interactions between factors. Inflammatory, hormonal, and genetic factors play a significant role in the development of PPD [9]. Additionally, psychological and social factors contribute substantially, with women who have a history of depression or anxiety disorders at higher risk of developing this condition [10,11]. Furthermore, studies have identified other important contributors, such as a family history of mood disorders, childbirth-related stressors, insufficient social support, and marital difficulties, all of which can increase the likelihood of PPD [12,13,14].

### 1.1. Social Support 

The influence of interpersonal relationships and social interactions on physical [15,16,17,18] and mental health [19,20,21] is well documented in the literature.

Social support encompasses both the structural aspects of individuals’ lives, such as group memberships or familial connections, and the functional roles these structures fulfill, particularly the provision of emotional and practical support [22,23]. It is essential to distinguish between perceived available support and actual received support [22,23]. Psychologists are particularly interested in perceived social support, which refers to an individual’s subjective assessment of the availability and adequacy of supportive resources from various sources, such as family, friends, and significant others [22,24]. This support can encompass emotional, informational, tangible/practical, and even belonging support [21,22,23].

Social support plays a critical role in shaping various outcomes during the childbirth process. It has been shown to reduce PTSD symptoms and aid in recovery from birth trauma [25,26,27], enhance breastfeeding self-efficacy [28], and promote greater parental role competence [29]. These findings underscore the importance of social support in supporting both maternal well-being and the transition to parenthood.

Specifically, in the context of PPD, perceived social support has also been recognized as a protective factor [30,31,32,33,34], even across different cultural contexts (e.g., Asia) [35]. Studies have consistently shown that mothers who perceive higher levels of social support are less likely to develop PPD symptoms, as social support can buffer against the stressors of motherhood and provide emotional resilience [36,37,38]. Social support plays a crucial role not only in reducing stress but also in enhancing a mother’s confidence in her parenting abilities and her sense of connection with others, both of which are essential for positive maternal mental health [29,30,39,40]. Some studies have explored not only the role of social support but also differential effects by family structure and source of support. Some authors have found that social support plays a protective role in postpartum depression, with intimate partner support being particularly important for married and cohabiting women [41], while other studies, such as Hughes et al. [42], suggest that support from friends can be equally significant in reducing psychological distress (i.e., depression and anxiety) for mothers during the transition to parenthood.

Conversely, the absence or inadequacy of social support has been associated with higher rates of PPD symptoms, as feelings of isolation and loneliness can exacerbate depressive symptoms [27]. Additionally, a lack of perceived social support during pregnancy has been associated with increased PPD symptoms, highlighting the importance of perinatal psychosocial factors in understanding postpartum psychological functioning [43,44,45]. This suggests that psychosocial variables throughout pregnancy play a critical role in shaping mental health outcomes after childbirth. In this sense, social support serves as both a protective factor against the development of PPD symptoms and as a buffer that mitigates the severity of symptoms in those already experiencing depression.

Although many studies have demonstrated the beneficial effects of social support on postpartum depression [31,32,33,35], the specific mediating mechanisms underlying this relationship are still not fully understood. Some research suggests that social support may reduce postpartum depressive symptoms by improving maternal self-efficacy [39,46,47], increasing the sense of competence [29,38], and reducing perceived stress [37,41]. In this study, we explored the role of emotion regulation difficulties.

### 1.2. Difficulties in Emotion Regulation

A growing body of research highlights the role of emotion regulation (ER) in mental health outcomes [48,49], particularly in depression [50,51]. In 1994, Thompson [52] described ER as the “extrinsic or intrinsic processes responsible for monitoring, evaluating, and modifying emotional reactions, especially their intensive and temporal features, to accomplish one’s goals” (pp. 27–28). Other authors [53] emphasize that ER refers to the processes individuals use to influence their emotions, including how they experience, express, and manage them to align with their goals. The same authors emphasize the use of several ER strategies that can be applied in different moments of the emotion generation sequence (e.g., situation selection, attention modification, or response modulation) [53].

ER difficulties specifically refer to challenges in managing and modulating emotional responses in a flexible and adaptive manner. These difficulties may include an inability to effectively identify, process, or control emotional states, leading to maladaptive emotional reactions, such as heightened emotional reactivity, impulsive behaviors in response to distress, or the avoidance of emotions. Individuals experiencing these difficulties often struggle to adjust their emotional responses to align with situational demands, which can contribute to the development or exacerbation of psychological disorders [54,55].

Particularly in the context of PPD, difficulties in ER have been recognized as a significant risk factor [56,57,58,59], as they impair a mother’s ability to manage the emotional and psychological demands of motherhood and diminish her capacity to respond sensitively to her baby’s needs, especially when they are distressed or dysregulated [60]. Additionally, interventions promoting ER for women after childbirth have been identified as effective in reducing postpartum depressive symptoms [61,62].

ER is not solely an individual psychological process—it is also shaped by external social factors, particularly social support. A growing body of literature suggests that the presence of strong social support networks can significantly enhance an individual’s capacity to regulate emotions effectively, particularly during periods of stress or emotional difficulty [63,64,65,66,67]. For instance, broader social networks provide increased opportunities for emotional expression, which in turn fosters more adaptable emotional responses [63]. Also, as proposed by some authors, social resources mitigate the effects of ER on depressive symptoms [68], suggesting that ER may be a significant pathway through which social support influences depression outcomes. In the context of PPD, these associations remained unexplored.

### 1.3. The Present Study

The present study aimed to examine the relationship between social support, ER difficulties, and PPD symptoms. A growing body of literature underscores the critical role of social support in enhancing individuals’ capacity to regulate emotions, while a lack of social resources has been shown to exacerbate ER difficulties [63,64,65,66,67]. Furthermore, ER has been identified as a key factor influencing mental health outcomes, including PPD [56,57,58,59].

Building on these findings, this study sought to explore how various forms of social support—particularly from partners, friends, and significant others—affect ER difficulties and in turn impact depressive symptoms during the postpartum period. By focusing on these relationships, this research contributes to a deeper understanding of how social resources and ER difficulties contribute to PPD symptoms. The anticipated findings are expected to inform future interventions aimed at improving mental health outcomes by integrating social support with strategies that enhance individual ER capacities to prevent or reduce PPD symptoms.

## 2. Materials and Methods

### 2.1. Participants

GPower software (version 3.1) was used for conducting power analyses to determine the appropriate sample size. GPower 3.1 is widely recognized for its accuracy and versatility in calculating statistical power across various test types, making it a reliable tool in psychological research. Recent research has continued to utilize GPower 3.1 for similar purposes, underscoring its relevance in contemporary studies (e.g., [40]). Sample size estimation was conducted for a multiple regression analysis with seven predictors (one independent variable and six mediators). Assuming a medium effect size (f^2^ = 0.15), an alpha level of 0.05, and a statistical power of 0.90, the required sample size was calculated to be 130 participants.

In this cross-sectional study, specific inclusion criteria were defined to ensure appropriate participant selection: (a) participants had to be at least 18 years old; (b) participants had to be within 12 months postpartum at the time of participation; and (c) participants had to provide informed consent. Exclusion criteria included: (1) recent history of severe psychiatric disorders (e.g., substance abuse or dependency), as determined by self-reports or diagnosed conditions, and (2) severe medical conditions such as cancer, as determined by self-reports. Data collection took place between January and August 2024 in Portugal.

A total of 160 women participated in the study, with ages ranging from 21 to 47 years (M = 33.57, SD = 4.94) and postpartum periods between 1 and 12 months (M = 6.79, SD = 3.94). Most of the participants were Portuguese (98.8%), married (86.3%), and had a higher education (69.9%). Participants’ characteristics are detailed in Table 1.

### 2.2. Measures

#### 2.2.1. Social Support Scale

Perceived social support was measured using the Multidimensional Scale of Perceived Social Support (MSPSS) [69]. The MSPSS (abbreviation commonly used) [70] was selected for assessing social support due to its comprehensive ability to measure support from multiple sources, including family, friends, and significant others (that can be combined to obtain a total score), and different types of support (e.g., emotional help, help to make decisions) [69]. The MSPSS has been extensively validated and widely used in research (e.g., [24,70]), demonstrating strong reliability and validity across various populations. Additionally, it is a brief self-report instrument, making it easy to administer and efficient for participants to complete.

The MSPSS comprises 12 items divided into three subscales (four items each) representing three sources of support: family (item example: “*I get the emotional help and support I need from my family*”), friends (item example: “*My friends really try to help me*”), and significant others (item example: “*There is a special person who is around when I am in need*”).

Each item is rated on a 7-point Likert scale, ranging from 1 (*Very strongly disagree*) to 7 (*Very strongly agree*). Higher scores indicate higher levels of perceived social support. The total score of the scale was used (Cronbach’s alpha = 0.94).

#### 2.2.2. Emotion Regulation Difficulties Scale

Emotion regulation (ER) difficulties were measured using the Difficulties in Emotion Regulation Scale—Short Form (DERS-SF) [71]. The DERS-SF (abbreviation commonly used) [57,58] was chosen to assess ER difficulties due to its strong psychometric properties and its ability to capture a wide range of difficulties in ER, our topic of interest [54]. This short form retains the core elements of the original DERS, while offering a more efficient and accessible measure for participants. The DERS-SF has been widely validated in both clinical and non-clinical populations, including in the context of postpartum depression [57,58].

The DERS-SF consists of 18 items measuring emotion regulation difficulties across six dimensions (each dimension with three items each): nonacceptance of emotional responses (item example: “*When I’m upset, I become embarrassed for feeling that way*”), difficulty engaging in goal-directed behavior (item example: “*When I’m upset, I have difficulty getting work done*”), impulse control difficulties (item example: “*When I’m upset, I become out of control*”), lack of emotional awareness (item example: “*I pay attention to how I feel*”), limited access to emotion regulation strategies (item example: “*When I’m upset, I believe that wallowing in it is all I can do*”), and lack of emotional clarity (item example: “*I have no idea how I am feeling*”).

Items are scored on a 5-point scale ranging from 1 (almost never, 0–10%) to 5 (almost always, 91–100%). Higher scores indicate more ER difficulties. The total score of the scale was used (Cronbach’s alpha = 0.87).

#### 2.2.3. Postpartum Depression Scale

Postpartum depression was measured using the Edinburgh Postnatal Depression Scale (EPDS) [72]. The EPDS (abbreviation commonly used) [35] was specifically designed to screen for postpartum depression and is widely regarded as one of the most reliable tools in maternal mental health research [31,32,35]. Given that our study focused on assessing depression during the postnatal period, the EPDS was the most suitable and well-established instrument for this purpose [31,32,35]. Its widespread use in numerous studies further enhances its reliability, making it an ideal tool for comparison with existing research. Additionally, the EPDS is a brief self-administered questionnaire, which ensures ease of use and allows participants to complete it quickly.

The EPDS consists of 10 items (item examples: “*I have been able to laugh and see the funny side of things*” and “*I have been anxious or worried for no good reason*”) that measure depressive symptoms in the last seven days. Each item is rated on a 4-point Likert scale, ranging from 0 to 3, with a total score ranging from 0 to 30. Higher scores indicate greater depressive symptoms. The EPDS showed good reliability in this study (Cronbach’s α = 0.89).

### 2.3. Procedure

Participants were recruited through a variety of channels to maximize outreach and ensure a diverse sample. Social media platforms played a significant role in disseminating information about the study, enabling researchers to reach a broader and more varied audience. Additionally, a gynecologist from a private clinic partnered with the study by informing patients about the research, offering them the opportunity to participate. All potential participants were provided with detailed information about the study’s purpose, objectives, and procedures to ensure they were fully informed.

Those who agreed to participate gave their informed consent prior to taking part in the study. Data collection was conducted using a comprehensive set of self-report questionnaires designed to gather relevant information. Participants had the option to complete the questionnaires in person, during a face-to-face interview at the clinic, or online, offering a flexible approach to accommodate their preferences and availability.

The study was thoroughly reviewed and approved by the Ethics Committee of Ispa—Instituto Universitário, ensuring compliance with established ethical standards for research involving human subjects. No incentives were provided to participants, aligning with the voluntary nature of their involvement and maintaining the integrity of the study. This meticulous process underscored the researchers’ commitment to ethical rigor and participant well-being.

### 2.4. Data Analysis

The collected data were analyzed using Statistical Package for the Social Sciences (SPSS) version 26. We used this version due to the available license for the researchers, and it continues to be commonly used in recent studies (e.g., [38]).

Descriptive statistics were computed to summarize demographic characteristics and main study variables. Pearson correlation coefficients were used to examine the associations between perceived social support, emotion regulation difficulties, and postpartum depression symptoms.

To test the hypothesized mediation model, the PROCESS macro (version 4.2) by Hayes [73] was utilized. PROCESS version 4.2 was selected for our analysis due to its comprehensive capabilities in modeling mediation, moderation, and conditional process models, which are central to our study’s objectives. Additionally, PROCESS is widely recognized for its ease of use, as it integrates seamlessly with SPSS, making it an ideal choice for our analysis. Moreover, it has been used in previous studies (e.g., [24,38]).

Specifically, model 4 of the PROCESS macro was used to examine whether emotion regulation difficulties mediated the relationship between perceived social support and postpartum depression. Thus, perceived social support was included as the independent variable, emotion regulation difficulties as mediators, and postpartum depressive symptoms as the dependent variable. The PROCESS macro estimates both the direct and indirect effects of the independent variable on the dependent variable through the mediator.

In this study, the indirect effect was tested using bootstrapping with 5000 resamples. A significant indirect effect was indicated if the 95% confidence interval for the indirect effect did not include zero. In addition to testing mediation, the R² value was used to assess the overall variance in postpartum depression explained by the model. Differences were considered significant at the level of *p* < 0.05.

## 3. Results

### 3.1. Preliminary Analysis

Descriptive statistics and correlations among study variables are presented in Table 2. Overall, perceived social support was significantly associated with fewer emotion regulation difficulties, ranging from −0.47 (limited access to strategies) to 0.39 (emotional awareness) and with fewer postpartum depressive symptoms. Emotion regulation difficulties were significantly associated with more postpartum depressive symptoms, ranging from 0.55 (non-acceptance of emotions) to −0.36 (emotional awareness).

### 3.2. Model Tested

Perceived social support was significantly associated with fewer postpartum depressive symptoms and fewer emotion regulation difficulties. Fewer emotion regulation difficulties were significantly associated with fewer postpartum depressive symptoms (except for lack of clarity and difficulty in impulse control).

**Figure 1:**
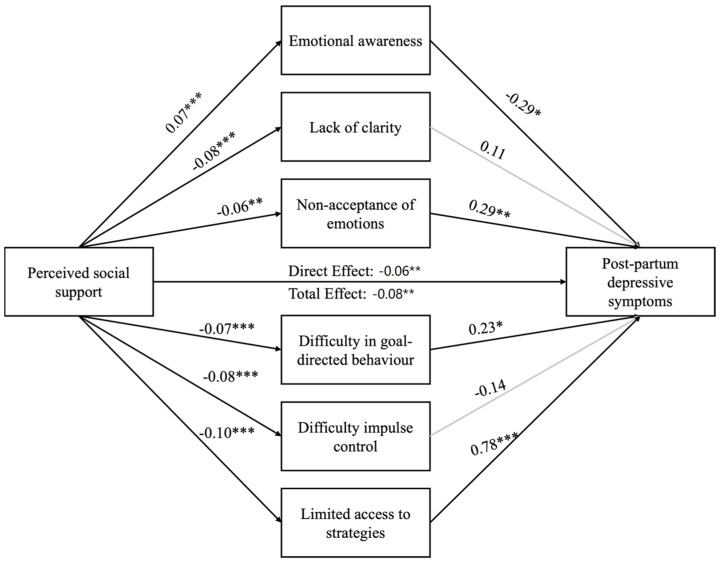
Associations between perceived social support, emotion regulation difficulties, and postpartum depressive symptoms (N = 160). Note: Gray lines indicate non-significant associations; * *p* < 0.05; ** *p* < 0.01; *** *p* < 0.001.

When the mediators were considered in the model, the link between perceived social support and postpartum depressive symptoms remained significant, suggesting that the associations between these two variables are partially mediated by most emotion regulation difficulties, namely, emotional awareness, non-acceptance of emotions, difficulty in goal-directed behavior, and limited access to strategies (see Figure 1). Indirect effects are reported in Table 3. The final model explained 61% of the postpartum depressive symptoms (F(7,152) = 33.68, *p* < 0.001).

## 4. Discussion

While having a child is generally a positive experience, the challenges faced during the postpartum period can sometimes lead to the development of PPD symptoms [74]. However, social support and effective emotion regulation can help mitigate these challenges, offering protective factors that may reduce the likelihood of developing PPD. Thus, the aim of this study was to investigate the relationship between social support, emotion regulation difficulties, and PPD symptoms.

First, as expected, we found a negative association between social support and PPD symptoms. These findings align with the existing literature [31,32,33,34,35,36], which suggests that women who perceive higher levels of social support are less likely to experience PPD symptoms. This protective effect of social support may be attributed to its role in enhancing maternal confidence and self-efficacy in parenting (e.g., [38,39,40]), as well as reducing feelings of isolation and loneliness [47,75], factors that can contribute to PPD symptoms. Additionally, social support can help reduce perceptions of stress, as having someone available to provide tangible or emotional support is particularly important during the postpartum period [37]. The presence of supportive individuals can alleviate the burden of daily responsibilities and emotional challenges related to parenting, offering a sense of security and reducing the overall stress experienced by new mothers.

We also found that emotion regulation difficulties were significantly associated with PPD symptoms, as expected [56,57,58]. This association underscores the critical role that the ability to manage and regulate emotions plays in postpartum mental health. Difficulties in emotion regulation can exacerbate negative emotional states and increase vulnerability to depressive symptoms. For example, individuals who struggle with regulating intense emotions may experience heightened stress and feelings of being overwhelmed in response to the challenges of postpartum life (e.g., sleep deprivation, caregiving demands).

Finally, we also found that certain emotion regulation difficulties partially mediated the relationship between social support and PPD symptoms, suggesting that social support may alleviate PPD symptoms in part by improving emotion regulation. These findings are consistent with the existing literature, which suggests that social resources and social support can significantly influence emotion regulation [63,67], thereby impacting psychological functioning.

However, not all types of emotion regulation difficulties played a significant role in mediating the relationship between social support and PPD symptoms. While social support was significantly associated with all types of difficulties, only difficulties in emotional awareness, acceptance of emotions, goal-directed behavior, and the use of effective regulation strategies were critical in explaining the link with PPD symptoms. These specific emotion regulation difficulties appear to be key mechanisms through which social support influences postpartum mental health outcomes. As previously highlighted by several authors, broader social networks offer more opportunities for emotional expression [63], which can enhance emotional awareness and acceptance by facilitating conversations about feelings and experiences. Additionally, social support can also facilitate the use of a broader repertoire of emotion regulation strategies. For instance, support providers can encourage individuals to engage in techniques such as distraction, cognitive reappraisal, or refocusing on positive aspects of a situation, strategies that individuals might otherwise struggle to implement on their own [66] and that can reduce PPD symptoms.

### 4.1. Limitations and Future Research

There are some limitations to this study that should be acknowledged. First, the relatively small sample may limit the generalizability of the findings, as it does not fully capture the diversity present in larger, more representative populations. This constraint highlights the need for caution when interpreting the results and applying them to broader contexts. Additionally, all participants were in the postpartum period, ranging from 1 to 12 months after childbirth. This variability in the timing of assessments could have influenced the consistency and reliability of the findings, as postpartum experiences and symptoms may differ significantly across this range.

The cross-sectional design of the study further limits the ability to establish causal relationships among social support, emotion regulation difficulties, and PPD symptoms. As such, the findings only provide a snapshot of these associations at a specific time point, leaving unanswered questions about their development and interactions over time. To address this limitation, future research employing longitudinal designs is necessary. Such studies would help confirm the directionality of these associations, shed light on how these factors evolve over time, and provide a more dynamic understanding of their interplay.

Moreover, the study did not account for several important variables that could have influenced PPD symptoms. Factors such as parental self-efficacy, stress management strategies, and broader psychosocial dynamics were not included in the analysis, limiting the comprehensiveness of the findings. By integrating these variables in future research, a more holistic understanding of the factors contributing to PPD could be achieved. Despite these limitations, the study provides valuable insights and serves as a foundation for further exploration in this critical area of maternal mental health.

Future research should aim to address these limitations by implementing more robust methodologies and expanding the scope of investigation. Larger, more diverse samples are essential to improve the generalizability of findings across different populations, considering variations in cultural, socioeconomic, and healthcare contexts. Employing longitudinal designs would allow researchers to track changes and interactions over time, providing insights into the temporal dynamics of PPD symptoms. Such designs should include an in-depth exploration of both stable personality traits (e.g., attachment styles) and perinatal variables (e.g., anxiety, depression, and fear of childbirth), as these factors are critical in shaping emotional and psychological responses during the perinatal period.

Furthermore, future studies should incorporate additional variables that are closely linked to PPD, but remain underexplored, such as individuals’ perceptions of traumatic birth experiences and their impact on mental health. Investigating these aspects could help uncover the complex interplay of biological, psychological, and social mechanisms that contribute to the onset and maintenance of PPD symptoms. By addressing these gaps, future research could not only advance theoretical understanding but also provide a stronger evidence base for developing personalized, preventative, and therapeutic interventions tailored to the needs of postpartum individuals.

### 4.2. Clinical Implications

The association between social support and reduced PPD symptoms highlights the importance of fostering and strengthening social networks for (new) mothers. Healthcare providers should assess the availability and quality of social support during postpartum visits, encouraging mothers to seek and maintain supportive relationships with partners, family, and friends. Interventions aimed at enhancing social support, such as partner or family counseling and peer support groups, could be beneficial in mitigating PPD symptoms. Given the key role of emotion regulation difficulties in PPD symptoms, interventions targeting emotion regulation skills may also be effective.

## Figures and Tables

**Table 1:** Participants’ characteristics.

Variable		%
Education	Basic education	6.3
	Secondary education	30
	Higher education	69.9
Marital status	Single	12.5
	Married or living together	86.3
	Divorced	1.3
Physical and/or mental health condition (self-reported)	Yes	16.3
	No	83.8
Time since birth	0–3 months	25.6
	4–6 months	25.7
	7–9 months	17.6
	10–12 months	31.3
Planned pregnancy	Yes	68.7
	No	31.3
High-risk pregnancy	Yes	44.4
	No	55.6
Average length of pregnancy	<37 weeks	10.0
	37–42 weeks	88.7
	>42 weeks	1.3
Type of birth	Vaginal	43.7
	Vaginal with forceps or vacuum	15.0
	Cesarean	41.3

**Table 2:** Descriptive statistics and correlations among study variables (N = 160).

	M (SD)	1.	2.	3.	4.	5.	6.	7.	8.
1. Perceived social support	58.19 (13.25)	-							
2. Emotional Awareness	11.76 (2.39)	0.39 **	-						
3. Lack of clarity	7.42 (2.85)	−0.35 **	−0.29 **	-					
4. Non-acceptance of emotions	8.09 (3.39)	−0.21 *	−0.21 *	0.41 **	-				
5. Difficulty in goal-directed behavior	9.66 (3.12)	−0.29 **	−0.09	0.40 **	0.43 **	-			
6. Difficulty in impulse control	7.26 (3.28)	−0.33 **	−0.22 *	0.47 **	0.49 **	0.46 **	-		
7. Limited access to strategies	7.49 (2.89)	−0.47 **	−0.23 *	0.57 **	0.61 **	0.56 **	0.64 **	-	
8. Depressive symptoms	9.81 (5.35)	−0.52 **	−0.36 **	0.50 **	0.55 **	0.51 **	0.46 **	0.71 **	-

Note: * *p* < 0.01; ** *p* < 0.001.

**Table 3:** Indirect effects of the links between perceived social support and postpartum depressive symptoms via emotion regulation difficulties (N = 160).

	Effect	SE	95%LL CI	95%UL CI
Emotional awareness	−0.02	0.01	−0.05	−0.00
Lack of clarity	−0.01	0.01	−0.03	0.01
Non-acceptance of emotions	−0.02	0.01	−0.04	−0.00
Difficulty in goal-directed behavior	−0.02	0.01	−0.04	−0.00
Difficulty in impulse control	0.01	0.01	−0.01	0.03
Limited access to strategies	−0.08	0.02	−0.12	−0.04

## Data Availability

Data presented in this study are available on request from the corresponding author. The data are not publicly available due to privacy and ethical restrictions.
